# A synchrotron computed tomography dataset for validation of longitudinal tensile failure models based on fibre break and cluster development

**DOI:** 10.1016/j.dib.2021.107590

**Published:** 2021-11-19

**Authors:** C. Breite, A. Melnikov, A. Turon, A.B. de Morais, C. Le Bourlot, E. Maire, E. Schöberl, F. Otero, F. Mesquita, I. Sinclair, J. Costa, J.A. Mayugo, J.M. Guerrero, L. Gorbatikh, L.N. McCartney, M. Hajikazemi, M. Mehdikhani, M.N. Mavrogordato, P.P. Camanho, R. Tavares, S.M. Spearing, S.V. Lomov, S. Pimenta, W. Van Paepegem, Y. Swolfs

**Affiliations:** a Department of Materials Engineering, KU Leuven, Kasteelpark Arenberg 44 box 2450, Leuven 3001, Belgium; b AMADE, Polytechnic School, University of Girona, Campus Montilivi s/n, Girona E-17003, Spain; c Department of Mechanical Engineering, RISCO research unit, University of Aveiro, Campus Santiago, Aveiro 3810-193, Portugal; d Universite de Lyon, INSA-Lyon, MATEIS, CNRS UMR5510, Villeurbanne F-69621, France; e Engineering Materials, Faculty of Engineering and Physical Sciences, University of Southampton, Southampton SO17 1BJ, UK; f INEGI, Rua Dr. Roberto Frias, 400, Porto 4200-465, Portugal; g Department of Engineering, Materials & Electrical Science, National Physical Laboratory, Teddington, Middlesex TW11 0LW, United Kingdom; h Department of Materials, Textiles and Chemical Engineering, Faculty of Engineering and Architecture, Ghent University, Technologiepark Zwijnaarde 46, Ghent, Belgium; i DEMec, Faculdade de Engenharia, Universidade do Porto, Rua Dr. Roberto Frias, Porto 4200-465, Portugal; j Department of Mechanical Engineering, Imperial College London, South Kensington Campus, London SW7 2AZ, United Kingdom

**Keywords:** Fibre-reinforced composites, Synchrotron computed tomography, Longitudinal tensile failure, Fibre breaks, Stress concentrations

## Abstract

We performed *in-situ* tensile tests on two carbon fibre/epoxy composites with continuous scanning using synchrotron computed tomography (CT). Both composites were cross-ply laminates, and two specimens were tested for each composite. The voxel size was sufficiently small to recognize individual fibres and fibre breaks. For each test, 16-19 volumes were reconstructed, cropped down to the 0° plies and analysed to track fibre break and cluster development. This dataset provides the last CT volume before failure for each of the four specimens as well as the individual fibre break locations in all reconstructed volumes. These data are then plotted against predictions from six state-of-the-art strength models. The target is that these data become a benchmark for the development of new models, inspiring researchers to set up refined experiments and develop improved models.

**Specifications Table**

**Table:** 

Subject	Ceramics and Composites
Specific subject area	Longitudinal tensile failure of fibre-reinforced composites analysed via synchrotron computed tomography and micromechanical modelling
Type of data	Table Image Figure
How data were acquired	For the SRCT data: • Synchrotron X-ray computed tomography at the TOMCAT beamline at SLS: • Source: 2.9-Tesla superbending magnet; Photon source size (h, v) = 140 µm, 45 µm (FWHM); Photon source divergence (tailored by aperture) (h, v) = 2 mrad (top-hat), 0.6 mrad (FWHM) • Scintillator: LuAG:Ce; Thickness = 20 µm • Microscope: High resolution white-beam microscope (Optique Peter) M Plan Apo 10x; Magnification = 10.0 (for 34-700) and 13.7x (for T700SC); • Detector: GigaFRoST (PSI in-house); Pixel size = 11.0 µm; Sensor size (h x v) = 2016 × 2016 px^2^ • Reconstruction: with an absorption-based algorithm provided by SLS For the modelling and comparison data: • The raw data was provided by the modelling participants based on the instructions included here and on the models they developed themselves • The averaging of the data was performed by KU Leuven
Data format	For the SRCT data: • 8 bit RAW files • Excel files For the modelling and comparison data: • Excel and PDF files
Parameters for data collection	Propagation distance = 60 mm for 34-700 and 170 mm for T700SC Beam energy = 20 kV Nr. of projections = 1000 for 34-700 and 1500 for T700SC Exposure time = 9 ms Voxel size = 1.1 μm for 34–700 and 0.8 µm for T700SC
Description of data collection	Double-edge notched specimens of carbon/epoxy laminate were scanned using synchrotron radiation computed tomography while being continuously loaded in tension.
Data source location	Institution: Swiss Light Source (SLS) at Paul Scherrer Institute (PSI) City/Town/Region: Villigen/Brugg Country: Switzerland Latitude and longitude (and GPS coordinates, if possible) for collected samples/data: 47°32′04.5″N 8°13′17.2″E
Data accessibility	Repository name: Mendeley Data Data identification number: 10.17632/6f2cv54ym8.1 Direct URL to data: https://data.mendeley.com/datasets/6f2cv54ym8/1
Related research article	C. Breite, A. Melnikov, A. Turon, A.B. de Morais, C. Le Bourlot, E. Maire, E. Schöberl, F. Otero, F. Mesquita, I. Sinclair, J. Costa, J.A. Mayugo, J.M. Guerrero, L. Gorbatikh, L. N. McCartney, M. Hajikazemi, M. Mehdikhani, M.N. Mavrogordato, P.P. Camanho, R. Tavares, S.M. Spearing, S.V. Lomov, S. Pimenta, W. Van Paepegem, Y. Swolfs, Detailed experimental validation and benchmarking of six models for longitudinal tensile failure of unidirectional composites, Composite Structures 279 (2022) 114828, DOI: j.compstruct.2021.114828.

**Value of the Data**

• The CT volumes constitute the most detailed dataset to date on fibre break and cluster development, which is vital for detailed experimental validation of current and future state-of-the-art models for longitudinal tensile failure.
• This data is useful for material scientists and mechanicians aiming to better understand the longitudinal tensile failure of composites, and using that knowledge to develop better materials.
• The users of this data can (1) use the fully processed information on fibre break and cluster development, (2) analyse the CT volumes to get additional information, for example on correlations between fibre break locations and local microstructure or (3) compare the model predictions against their own predictions.

## 1 Data Description

The data presented in this article is available as a Mendeley data set [1]. It contains three parts:

• The detailed instructions for the participants, including all required material data.
• The synchrotron radiation computed tomography (SRCT) data
• The processed experimental data and plots to compare it with model predictions

The detailed instructions for the participants contains 11 files:

• “*3M_Scotch-Weld EC-9323 BA datasheet.pdf*”: datasheet of the glue used for gluing the end tabs onto the panels.
• “*34-700 carbon fibre datasheet.pdf*”: datasheet of the 34-700 carbon fibres.
• “*Background of matrix characterisation.pdf*”: a detailed explanation of how the neat matrix characterisation was performed.
• “*Details of benchmarking exercise II - Instructions for participants.pdf*”: detailed instructions describing the goals, materials, methods and modelling requirements.
• “*Extra input for benchmarking exercise.pdf*”: description of how the stress redistributions from the models shown in the parent paper were set up.
• “*Fibre strength and diameter measurements.xlsx*”: all individual measurements of fibre strength and diameters, which were used to set up the Weibull distributions for fibre strength.
• “*KTA315_20C_0001_1_s_Material_Data.xlsx*”: results of the characterisation of the SR8500/KTA315 resin system.
• “*736LT_20C_0001_1_s_Material_Data.xlsx*”: results of the characterisation of the 736LT resin system.
• “*NTPT ThinPreg 736LT datasheet.pdf*”: datasheet for the commercial prepreg.
• “*SiPreg SR 8500 KTA 31x data sheet.pdf”:* datasheet for the SR8500/KTA315 resin system.
• “*T700S carbon fibre datasheet.pdf*”: datasheet for the T700SC carbon fibre.

The SRCT data contains the following parts:

• The 8-bit RAW files constituting the last volume before failure for each of the four tested specimens. Other volumes can be obtained from the corresponding author.
• Four Excel files describing the coordinates of all fibre breaks and clusters in every reconstructed volume for each of the four tested specimens. All the reconstructed volumes were registered to the same location, so that the coordinates of the fibre break stay more or less consistent between the volumes.

The processed experimental data:

• “*Orientation_analysis_summary.xlsx*”: summary of the analysis of the fibre orientations for all four specimens.
• “*Residual thermal strains calculations.xlsx*”: the calculations of the residual thermal strains that are used to correct the macroscale tensile tests.
• “*Results - basics.xlsx*”: analysis of the computational time, failure strain, strength, stress-strain diagrams, fibre break density and largest cluster development.
• “*Results - cluster height and stdev.xlsx*”: analysis of the development of the cluster height and the cluster height standard deviation.
• “*Results - macroscale tensile tests.xlsx*”: the results of the macroscale tensile tests.
• “*Results - plet evolution.xlsx*”: analysis of the cluster development.
• “*Results - stress redistribution.xlsx*”: comparison of the stress redistributions of the models for a single fibre break and two coplanar fibre breaks.
• “*Results - variability analysis.xlsx*”: analysis of the variability in the model predictions and microscale experiments for fibre break density, largest cluster, 2-plet density and 3-plet density.
• “*Results - Weibull scatter analysis with HSL for 34700 case.xlsx*”: analysis of the influence of the uncertainty on the Weibull strength distribution on the predictions for the 34-700 case using the HSL model.
• “S*tress back-calculation and Vf normalisation for macroscale tests.xlsx*”: an example of how the stress back-calculation and fibre volume fraction normalisation was performed on the macroscale tests.

## 2 Experimental Design, Materials and Methods

This study investigated two different types of materials. The first material was a commercial prepreg from North Thin Ply Technology (Switzerland) with Grafil 34-700WD-24K-1.4%A carbon fibres (Mitsubishi Chemical) and proprietary 736LT resin. Their measured fibre areal density and cured ply thickness were approximately 38 g/m^2^ and 44 µm, respectively. The second material was based on T700SC-12K-50C carbon fibres (Toray Europe, France) and SiPreg SR8500-KTA315 epoxy resin (Sicomin, France). These T700SC fibres were drum wound with the epoxy resin at KU Leuven to obtain prepregs with a measured fibre areal density of about 172 g/m^2^, and a cured ply thickness of 191 µm. These materials are referred hereafter to as “34-700” and “T700SC”, respectively.

In addition, two commercial glass fibre prepregs were sourced. The first prepreg was from North Thin Ply Technology (Switzerland) and contained HYBON-2026 E-glass fibres and 736LT resin. These prepregs had a nominal fibre areal density of 50 g/m^2^, an average cured ply thickness of 34 µm and a fibre volume fraction of 58%. The second prepreg was from Hexcel (UK) and contained SCG75 S-2 glass fibres and 913 epoxy matrix. These prepregs had a nominal fibre areal density of 190 g/m^2^, an average cured ply thickness of 150 µm and a fibre volume fraction of 60%. This material was not studied here, but used in the outer layer of the macroscale specimens to avoid stress concentration as explained below.

Specimens for microscale and macroscale tests were manufactured. For the microscale specimens, ${\left[90_{4}^{C}/0_{4}^{C}\right]}_{s}$ and ${\left[90^{C}/0^{C}\right]}_{s}$ layups were manufactured for the 34-700 and T700SC materials, respectively. For the macroscale specimens, ${\left[0_{16}^{EG}/90_{4}^{C}/0_{4}^{C}\right]}_{s}$ and ${\left[0_{5}^{SG}/90^{C}/0^{C}\right]}_{s}$ hybrids layups were manufactured for the 34-700 and T700SC materials, respectively. The superscripts SG, EG and C stand for S-glass, E-glass and carbon layers, respectively. The presence of the glass layers eliminated the stress concentrations on the carbon layers near the grips, as demonstrated by Wisnom, Czél et al. [2,3]. This enables failure in the gauge section, and hence a reliable determination of the failure strain.

Laminates of 300 × 300 mm^2^ were manually stacked, and then cured in KULeuven's autoclave. The curing cycle followed the manufacturer's recommendations. For the 34-700 laminates, the laminate was heated from room temperature to 70°C at 2°C/min. After 60 min, the temperature was increased to 120°C at a heating rate of 1.4°C/min. After 45 min, the autoclave was then cooled down to room temperature at 1.4°C/min. A vacuum pressure of -0.7 bar was applied during the entire cycle, and the overpressure of 5 bar applied from the moment 70°C was reached and maintained until the end of the curing cycle. The T700SC laminate followed a similar curing cycle: the only differences were in the heating rates (2.8 and 1.4°C/min) and the dwell time at 120°C being 90 min rather than 45 min.

For the macroscale tests, a water-cooled diamond saw was used to cut rectangular, parallel-sided specimens without a notch from the cured panels. The ASTM D3039 standard was followed to perform tensile tests on a Zwick Z100 universal testing machine at KU Leuven with a 100 kN load cell. The gauge length was 150 mm for the 34-700 specimens, respectively whereas this was 170 mm for the T700SC specimens. The nominal specimen width was 16 mm. 4 mm thick woven E-glass epoxy composite end tabs were glued onto the tensile specimens. The length was 50 and 40 mm for the 34-700 and T700SC specimens, respectively. The displacement rate was 0.5 mm/min for the seven 34-700 specimens, whereas it was 1 mm/min for the ten T700SC specimens. The longitudinal surface strain was measured using an optical extensometer for the 34-700 specimens, whereas digital image correlation was used for the T700SC specimens.

The presence of the glass fibre layers necessitates a back-calculation to measure the stress in the 0° carbon plies, and a correction for the thermal residual stresses. This is elaborated in more detail in the dataset file “S*tress back-calculation and Vf normalisation for macroscale tests.xlsx*”. In addition, the strength and stress values were normalised to the modelled V_f_.

For the microscale specimens, double-notched specimens were cut from the cured panels using water jet cutting. 1 mm thick aluminium tabs were glued to the specimen ends using 3M Scotch-Weld EC 9323 B/A. The glue was cured in an oven at 100°C for 15 min for the 34-700 specimens and at 60°C for 60 min for the T700SC specimens.

The TOMCAT beamline at Swiss Light Source (SLS) was used to perform *in-situ* SRCT. The measurements were performed using the INSA Lyon tension-compression rig. The displacement rate was selected to achieve failure within approximately 7-10 min. The GigaFRoST camera was used to enable continuous scanning [4]. Absorption-based reconstruction was performed using the in-house algorithms supplied by SLS. A total of 16-19 volume were reconstructed for each of the four specimens. The scans showed a relatively uniform fibre distribution, a low void content and good overall specimen quality. While water jetting introduced some tapering, no other damage was visible at the specimen edge. The notch and accompanying stress concentrations were effectively removed when splits occurred at 55–65% and 40-50% of the failure load for the 34-700 and T700SC specimens, respectively. Table 1 in the related research article [5] summarises the details of the SRCT scan settings.

The fibre alignment of the specimens were analysed using VoxTex, and the results are summarised in the dataset file *Orientation_analysis_summary.xlsx*. The void content was also analysed, but was found to be so low that it could not be accurately quantified.

The SRCT volumes were slightly rotated to have the same laminate coordinate system for all specimens. The volumes were then cropped down to the volume of interest, being the 0° fibres that are continuous over the notch. The presented dataset [1] provides the last volume of interest before failure for each of the four specimens. The first step in counting fibre breaks was a detailed manual count of the fibre breaks in the last volume before failure. Once this was completed, the same fibre breaks were sought in preceding volumes to detect when they first appeared. All fibre break coordinates were noted down for every volume, and it was then checked whether they belong to a cluster. Fibre breaks were assumed to be a cluster if they were within an axial distance of fifteen fibre diameters and a centre-to-centre radial distance of two fibre diameters. An n-plet is defined as a cluster containing ‘n’ fibre breaks. For stress calculations, the cross-sectional area of the 0° ply in the last volume before failure was measured using FiJi's polygon tool [6]. The last volume may have a slightly smaller cross-sectional area than the first volume due to Poisson's contraction, but the first volume could not be used for cross-sectional area calculations because the splits had not occurred yet. The stress was defined as the load divided by the 0° cross-sectional area, hence ignoring the stress contribution of the 90° plies. This contribution is limited due to their low stiffness relative to the stiffness of the 0° plies, and even further reduced by the presence of transverse cracks and delaminations.

The fibre volume fraction V_f_ was measured by counting the number of individual fibres in a cross-section using the InSegt Fibre algorithm developed by Emerson et al. [7]. Combining this information with the fibre diameter and cross-sectional area measurements, the V_f_ could be calculated. The standard deviation on the V_f_ was obtained by performing this calculation on about 30 cross-sections.

Single fibre tensile tests were performed using the LEX/LDS automated tester at Dia-Stron Limited [8]. All fibre diameter and strength values are summarised in the dataset file *Fibre strength and diameter measurements.xlsx.* 89 and 92 measurements were performed for the 34-700 and T700SC fibres, respectively. The parameters of the unimodal Weibull distribution (summarised in Table 3 of the related research article [5]) were obtained using the maximum likelihood estimator.

Most of the elastic constants and thermal expansion coefficients are based on the literature [3,[9], [10], [11], [12]–13]. The longitudinal modulus E_11_ was taken directly from the manufacturer's datasheet for the carbon fibres, whereas it was based on the literature for the glass fibres [13,14]. The Weibull parameters, σ_0_, m, L_0_, are taken from the abovementioned single fibre tensile tests [8].

The stress-strain response of the epoxy resins was characterised using the methodology developed by Morelle et al. [15]. This methodology is described in more detail in the dataset file *Background of matrix characterisation.pdf*. The tensile modulus was 3.15 GPa and 3.36 GPa for the 736LT and SR8500 epoxy resins, respectively. The shear onset was suggested as the best estimate for the shear yield stress in the models using perfect plasticity, and was in fact used by all participating models. This parameter was defined as the maximum shear stress prior to softening, and was 60.4 and 63.3 MPa for 736LT and SR8500, respectively. The same tests also yielded the Poisson's ratio for the epoxy resins: 0.39 and 0.42 for 736LT and SR8500, respectively. The literature [3,^11^,12] was used to estimate 62.5 10^−6^/K for the CTE of both epoxies.

Table 5 of the related research article reveals the fibre volume fractions, geometries and fibre counts for all specimens. The minor discrepancies in fibre volume fraction between the microscale, macroscale and modelled specimens were corrected for by linearly normalising stress and strength values to the modelled V_f_.

The experimental data in the dataset is compared to the predictions of six different state-of-the-art models. Since these models have already been described in detail in the literature, their details will not be described here. Table 3 of the related research article provides an overview of these six models, as well as the key references describing the models. 3PFM and FEISM submitted 50 Monte Carlo run, whereas ABS and DYSEM submitted 10 Monte Carlo runs. DFBM and HSL only submitted the statistically expected values, as they are probabilistic by nature.

## Ethics Statement

This work has not involved any use of human subjects and animal experiments.

## CRediT authorship contribution statement

**C. Breite:** Conceptualization, Methodology, Software, Formal analysis, Investigation, Writing – review & editing. **A. Melnikov:** Conceptualization, Methodology, Software, Formal analysis, Investigation, Writing – review & editing. **A. Turon:** Writing – review & editing, Supervision. **A.B. de Morais:** Software, Investigation, Writing – review & editing. **C. Le Bourlot:** Methodology, Investigation, Writing – review & editing. **E. Maire:** Methodology, Investigation, Writing – review & editing. **E. Schöberl:** Conceptualization, Methodology, Investigation, Writing – review & editing. **F. Otero:** Software, Writing – review & editing. **F. Mesquita:** Conceptualization, Methodology, Software, Investigation, Writing – review & editing. **I. Sinclair:** Conceptualization, Methodology, Investigation, Writing – review & editing, Supervision. **J. Costa:** Writing – review & editing, Supervision. **J.A. Mayugo:** Writing – review & editing, Supervision. **J.M. Guerrero:** Software, Investigation, Writing – review & editing. **L. Gorbatikh:** Writing – review & editing, Supervision. **L.N. McCartney:** Software, Investigation, Writing – review & editing. **M. Hajikazemi:** Software, Investigation, Writing – review & editing. **M. Mehdikhani:** Formal analysis, Investigation, Writing – review & editing. **M.N. Mavrogordato:** Methodology, Writing – review & editing, Supervision. **P.P. Camanho:** Writing – review & editing, Supervision. **R. Tavares:** Software, Investigation, Writing – review & editing. **S.M. Spearing:** Conceptualization, Methodology, Investigation, Writing – review & editing, Supervision. **S.V. Lomov:** Writing – review & editing, Supervision. **S. Pimenta:** Conceptualization, Methodology, Software, Investigation, Writing – review & editing. **W. Van Paepegem:** Investigation, Writing – review & editing, Supervision. **Y. Swolfs:** Conceptualization, Methodology, Software, Formal analysis, Investigation, Writing – original draft, Visualization, Supervision.

## Declaration of Competing Interest

The authors declare that they have no known competing financial interests or personal relationships which have, or could be perceived to have, influenced the work reported in this article.
